# Mechanisms of ARIA: is it time to focus on the unique immune environment of the neurovascular unit?

**DOI:** 10.1186/s13024-023-00667-8

**Published:** 2023-10-20

**Authors:** Kate E. Foley, Erica M. Weekman, Donna M. Wilcock

**Affiliations:** 1 Stark Neurosciences Research Institute, Indiana University School of Medicine, 320 W 15th St, Indianapolis, IN 46202 USA; 2 Department of Neurology, Indiana University School of Medicine, Indianapolis, IN 46202 USA; 3 Anatomy, Cell Biology and Physiology, Indiana University School of Medicine, Indianapolis, IN 46202 USA

**Keywords:** ARIA, Amyloid, Immunotherapy, Cerebrovasculature, Blood brain barrier, Perivascular macrophages, Microglia

Microhemorrhages as a consequence of anti-Ab immunotherapy were first identified over 20 years ago in a single page report in Science by Pfeifer et al. [1]. Following on from that, both Wilcock and Racke reported microhemorrhages in 2004 and 2005 respectively, in different animal models and with different Ab antibodies [2, 3]. When vasogenic edema and microhemorrhages were identified in clinical trials of bapineuzumab in 2009, a working group coined the term “amyloid related imaging abnormalities” (ARIA) [4]. ARIA-E refers to vasogenic edema and ARIA-H refers to hemorrhagic events including microhemorrhages. With the FDA approval of anti-Ab immunotherapies, ARIA continues to plague these therapies and remains the major adverse event limiting the broad use of these therapies. It is now more important than ever that we as a field work to understand the underlying mechanisms of ARIA so that we can develop next generation immunotherapies and adjunct treatments to mitigate the incidence and severity of ARIA events. In the recent publication by Taylor et al., the researchers move us closer to understanding the mechanisms of microhemorrhages due to anti-Ab antibodies [5].

Taylor et al. first established an anti-Aβ 3D6 (murine N-terminal anti-Ab antibody)-dependent reduction in plaque, increase in microhemorrhages, and altered leptomeningeal vessel diameter in 23-month-old PDAPP mice treated through weekly intraperitoneal injection for three months. Since the seminal paper from Keren-Shaul delineating different microglia states in an amyloidogenic mouse model, dozens of other microglia enthusiasts have fought to identify their own ‘subtype,’ however, microglia have been shown not to respond as voraciously to vascular amyloid as they do to parenchymal plaques [6]. Understandably, Taylor et al. investigated microglia in the current study. Prior studies have suggested an association between microglial engagement and microhemorrhage incidence [7, 8]. This finding has been reproduced through no association between P2Y12 + microglia and DAM marker, Clec7a + microglia with CAA in 3D6 treated animals. The major finding in this work is the involvement of the long-forgotten cousin of the microglia, the perivascular macrophage.

Immunofluorescent staining for macrophages (Mac387), and more specifically, perivascular macrophages (CD169), show significant colocalization in both leptomeningeal and penetrating vessels. This finding was further supported by CD206 and CD163 perivascular staining after one month of 3D6 injections in another amyloidogenic mouse model exhibiting CAA. However, it was unclear which of the two mouse models in the reference paper was used (APP NL-F, or APP NL-G-L). The important difference between these models being the presence or absence of the Arctic mutation which increases Aβ40 production, which is the primary Aβ isoform in vascular amyloid, or CAA. Hypothesizing that the triggering of the Fc receptors influences the perivascular macrophage phenotype, the team then evaluated transcriptional changes in bone marrow derived macrophage (BMDM) cultures plated with intact IgG2a or an IgG2a with an ‘effector-silent’ mutation in the Fc portion [9]. Interestingly, gene expression analysis via the Nanostring neuroinflammation panel, revealed BMDMs increased matrix remodeling genes in the matrix metalloproteinase family (MMP), such as Timp1, Mmp14, and Mmp12. Activation of the MMPs has been associated in stroke with events such as hemorrhagic transformation and pathologic angiogenesis [10]. In fact, the finding of Taylor et al. aligns with our own prior studies that found increased MMP2, MMP3, MMP14, and MMP9 in two distinct immunotherapy studies in two different mouse models [11].

Overall, the data from Taylor et al. suggests that macrophages, when exposed to immune complexes, may contribute to blood brain barrier integrity through regulation of the basement membrane. The other top hits revolved around ‘inflammatory signaling’, ‘cytokine signaling’, ‘Nf-kB signaling’ and ‘innate immune response’. Taylor et al. further verifies protein upregulation of Timp1, an MMP inhibitor, and MMP9, which was increased in perivascular macrophages in response to 3D6 in the PDAPP mice. This finding suggests that the stabilization of the basement membrane around leptomeningeal and penetrating vessels, which are typically the arteries that reflect the heaviest burden of CAA, may be a possible avenue to mitigate ARIA. Notably, there are several FDA-approved drugs targeting MMP activity exist due to MMP involvement in cancerous tumor growth and angiogenesis, however, the long-term effects of MMP inhibition could be consequential. Alternatively, targeting pathways upstream of MMP expression may offer an alternative approach. What is unclear, however, is how potential inhibition of these ARIA-related pathways may impact efficacious amyloid removal, since it has become increasingly apparent that microglial engagement is necessary for amyloid lowering.

Overall, the publication from Taylor and colleagues provides unique insights into the potential mechanisms of ARIA. Future studies will need to be pursued to determine whether efficacy and ARIA can be disconnected in some way so that amyloid lowering can continue while mitigating blood-brain barrier breakdown leading to ARIA.

*************************

Response from Dr. Ronald B. Demattos on the behalf of the authors of 2023 *Mol Neurodegener* paper in Ref 4:

We would like to thank Drs Foley, Weekman, and Wilcock for their research highlight of the Taylor et al. manuscript as well as the opportunity to provide clarifying details for one of the animal models used in the original work. They very astutely identified that additional details pertaining to the hAPP-KI/hTau would be beneficial. The hAPP-KI/hTau mice were generated as follows:

The Lilly APP Knock-In (APP KI) mice were generated by knocking into the mouse APP gene the Swedish and Iberian mutations (K670N, M671L, I716F) and humanized Aβ domain (G676R, F681Y, R684H), a similar approach as that used previously to generate the APP NL-F (Saito et al. Nat. Neurosci 2014). Note, the Lilly APP KI mice do not express the Artic mutation. The APP KI animals were subsequently crossed to hTau mice (Andorfer et al. J. Neurochem 2003) to generate the hTau/APP mice that are homozygous for humanized APP but hemizygous for human genomic MAPT on mouse tau knockout background.

******************************************

## Data Availability

Not applicable.

## Abbreviations

Aβ: Amyloid Beta
AD: Alzheimer’s Disease
ARIA: Amyloid Related Imaging Abnormalities
BMDM: Bone Marrow Derived Macrophage
CAA: Cerebral Amyloid Angiopathy
FDA: Food and Drug Administration
MMP: Matrix Metalloproteinase
